# Effectiveness of office hysteroscopy for retained products of conception: insights from 468 cases

**DOI:** 10.1007/s00404-025-08075-7

**Published:** 2025-05-31

**Authors:** Giosuè Giordano Incognito, Katja Jakopič Maček, Mija Blaganje, Kristina Drusany Starič, Giuseppe Ettore, Carla Ettore, Maša Lukež Podgornik, Ivan Verdenik, Nataša Kenda Šuster

**Affiliations:** 1 Obstetrics and Gynecology Unit, Maternal Child Department, Garibaldi Nesima Hospital, Via Palermo 636, 95122 Catania, Italy; 2 Department of Gynecology and Obstetrics, University Medical Centre Ljubljana, Zaloška cesta 2, 1000 Ljubljana, Slovenia; 3 Medical Faculty, University of Ljubljana, Vrazov trg 2, 1000 Ljubljana, Slovenia

**Keywords:** Minimally invasive surgery, Office hysteroscopy, Retained products of conception, Uterine diseases

## Abstract

**Purpose:**

Retained products of conception (RPOC) are a common complication following pregnancy. Office hysteroscopy (OH) is increasingly used for diagnostics and management due to its minimally invasive nature. However, incidence of incomplete OH removal and procedures in which no RPOC are identified despite prior suspicion remains a concern. This study aimed to identify factors associated with these outcomes to improve patient selection and procedural success.

**Methods:**

A retrospective study was conducted on patients referred for OH for presumed RPOC between August 2015 and April 2023 at the Department of Gynecology and Obstetrics, University Medical Centre Ljubljana, Slovenia. Inclusion criteria included hemodynamically stable patients with prolonged bleeding and/or suspicious ultrasound (US) findings post-pregnancy. Patients with RPOC thickness of more than 30 mm or strong tissue vascularization on US were excluded. Data on patient demographics, US features, and procedural outcomes were analyzed.

**Results:**

Out of the 468 patients, RPOC removal was performed in 333 cases (71.2%), of which 225 (67.6%) were successfully completed, while in 135 cases (28.8%), the procedure was only diagnostic due to the absence of RPOC. Regarding procedural success, neither the pregnancy outcome, i.e., termination of pregnancy (TOP) vs delivery, nor gestational age significantly correlated with it. Longer time from pregnancy end to OH significantly improved procedural success in both groups, after TOP (*p* = 0.025) and in cases of large RPOC after delivery (*p* < 0.001). Parity significantly altered procedural success only in the delivery group (*p* < 0.007). The success rate of the procedure was significantly higher in cases when only small RPOC were observed (*p* < 0.001). Absence of RPOC occurred more frequently following TOP than after delivery (*p* < 0.001). Procedures in which no RPOC were confirmed were significantly associated with a longer interval between pregnancy end and OH in both TOP (*p* = 0.013) and delivery group (*p* = 0.003). Gestational age significantly correlated with the absence of RPOC only in the delivery group (*p* = 0.003). The likelihood of not confirming RPOC was higher where US thickness and length were lower (*p* = 0.007 and *p* = 0.011, respectively).

**Conclusion:**

OH is effective for managing RPOC with a high success rate, but the absence of RPOC in a considerable number of OH-treated patients stresses the need for better diagnostic criteria and patient selection to minimize overtreatments. Further prospective studies are needed to optimize the timing and indications for OH.

## What does this study add to the clinical work

**Table:** 

Office hysteroscopy offers a high success rate in selected patients with suspected retained products of conception	
A combination of preoperative and intraoperative factors can help anticipate procedural outcomes and guide clinical strategies.	

## Introduction

Retained products of conception (RPOC) are defined by the persistence of trophoblastic or placental tissue in the uterine cavity after a miscarriage, abortion, or delivery [1]. Although the exact prevalence remains unclear, this complication is estimated to affect approximately 1–5% of pregnancies [2]. RPOC is associated with a broad spectrum of clinical scenarios, including abnormal uterine bleeding, pelvic pain, infections, and fever [3]. Transvaginal ultrasonography (US) is generally considered the first-line diagnostic tool for evaluating women suspected of having RPOC. It typically reveals a heterogeneous intracavitary hyperechoic focal mass, often accompanied by a poorly defined interface between endometrium and myometrium and/or increased, irregular endometrial thickness. In less common cases, a polypoid or pedunculated mass of placental tissue (placental polyp) may be observed [4, 5]. The use of color Doppler US is recommended to enhance diagnostic accuracy. The presence of vascularization in the RPOC or the underlying myometrium indicates stronger tissue vascularization, which is associated with an increased risk of significant bleeding during OH removal [6]. Imaging abnormalities at the tissue–myometrial border may suggest placentation abnormalities or invasion into the myometrium [7]. However, the diagnostic accuracy of US for RPOC varies significantly among studies, highlighting the need for cautious interpretation of findings [8, 9]. Dilation and curettage (D&C), often performed with vacuum aspiration and under US guidance, has long been considered the standard surgical technique for removing RPOC. However, it is a blind procedure and carries the risk of several complications, including incomplete evacuation of RPOC and uterine perforation [10, 11]. Additionally, the non-selective tissue removal can damage the endometrium’s basalis layer, potentially resulting in the development of intrauterine adhesions, commonly known as Asherman’s syndrome, which can significantly impair reproductive outcomes [12]. Hysteroscopy is widely regarded as the gold standard for diagnosing and managing intrauterine disorders. It offers several advantages over the traditional method, including direct visualization of the uterine cavity, selectively removing tissue without damaging the surrounding healthy endometrium, and a reduced risk of complications during the procedure [13]. Historically, diagnostic hysteroscopy has been recommended to confirm US findings [14]. When treatment is required, operative hysteroscopy is typically performed under general anesthesia to minimize pain and enable the use of advanced hysteroscopic instruments, including those that employ electricity [15]. The advent of small-diameter hysteroscopes with operative channels has facilitated single-stage “see-and-treat” hysteroscopy (OH). This approach allows diagnostic and operative procedures to be performed simultaneously in an outpatient setting, resulting in significant cost- and time-savings while eliminating the need for general anesthesia, operating rooms, and hospitalization [16]. Furthermore, the introduction of the vaginoscopic approach by Bettocchi et al. has revolutionized the procedure by eliminating the need for a tenaculum and speculum, thereby avoiding cervical dilation and enhancing patient comfort [17]. Nevertheless, the smaller instruments commonly used in OH can present significant limitations, mainly when dealing with larger masses, tissues firmly adhered to the myometrium, or cases involving pronounced bleeding [18]. Despite these challenges, OH has become a widely adopted technique for diagnosing and treating many intrauterine pathologies. However, there has been limited advancement in the literature regarding its role in managing RPOC since a previous work by this group [19]. It is also crucial to consider that, even when RPOC is diagnosed, OH may fail to achieve complete removal. Furthermore, the procedure may occasionally be performed for suspected RPOC but result in no intrauterine remnants being identified, leading to avoidable patient discomfort and resource utilization. In light of these considerations, the present study aimed to identify the factors associated with the incomplete removal of RPOC and with the absence of RPOC during OH. By addressing these limitations, the study’s scope was to enhance the understanding and effectiveness of OH in this clinical context, ultimately improving patient outcomes and optimizing the use of healthcare resources.

## Materials and methods

### Study design and setting

This retrospective study aimed to analyze data of patients with presumed prolonged RPOC who were referred to an OH from August 2015 to April 2023 at the Department of Gynecology and Obstetrics, University Medical Centre Ljubljana, the largest tertiary care center in Slovenia. This study was approved by the Republic of Slovenia National Medical Ethics Committee on 17.10.2023 (approval number: 0120-265/2023/3) and conducted in accordance with the principles of the Declaration of Helsinki. OH was performed in an outpatient setting without cervical dilation or anesthesia using either 5.5 or 4.2 mm rigid hysteroscopes (Karl Storz®, Tuttlingen, Germany) or the Truclear 5.2 mm Tissue Removal System (Medtronic®, Minneapolis, USA). A vaginoscopic approach was used, so no speculum or tenaculum was applied.

### Participants

The inclusion criteria were hemodynamically stable patients with no signs of infection, with prolonged bleeding and/or suspicious US findings after termination of pregnancy (TOP) or postpartum. We defined TOP as pregnancy termination (spontaneous or after medical intervention) before 22 weeks of gestation and delivery beyond this threshold. Patients with a high risk for surgical complications (RPOC thickness measured on transvaginal US of more than 30 mm or strong tissue vascularization assessed by power Doppler on transvaginal US) were excluded.

### Variables

The following preoperative findings were retrieved: patient age, previous deliveries, mode of TOP (medical, surgical, or spontaneous) or delivery (vaginal or cesarean), gestational age, D&C attempts resulted in incomplete removal of RPOC, US thickness and length of suspected RPOC, the shape of borders between RPOC and the myometrium on US (classified as linear, unclear, or creased), and vascularization of the tissue and directly underlying myometrium estimated with power Doppler (described as absent or minimal in accordance with IETA color scores 1 and 2 as described by Leone et al. [20]).

The intraoperative and postoperative data noted included the time from the end of pregnancy to OH, type of procedure (diagnostic or removal), RPOC dimensions on OH (categorized according to the internal protocol as “small” when less than one half of uterine cavity volume was involved, “large” when more than one half of uterine cavity volume was involved, or absent when there were no remnants or just a trace of them), duration of the procedure, surgeon’s estimation of procedure success (described as complete or incomplete), complications during the procedure, patient pain assessment after the procedure on a visual analog scale (VAS) from 1 to 10, and the need for subsequent hysteroscopy or D&C during follow-up. Patients’ clinical data were recorded and stored in the electronic health record database. All information was extracted anonymously, ensuring privacy and compliance with ethical standards. This study was approved by the Republic of Slovenia National Medical Ethics Committee on 17.10.2023 (approval number: 0120-265/2023/3) and was conducted in accordance with the principles of the Declaration of Helsinki.

### Statistical methods

Statistical analyses were performed using IBM SPSS Statistics (version 29.0, IBM Corp., Armonk, NY, USA). Continuous variables were presented as means and standard deviations (SD), while categorical variables were reported as frequencies and percentages. Comparisons of continuous variables between groups were conducted using Student’s *t* tests for independent samples or one-way analysis of variance (ANOVA) as appropriate. Bonferroni correction was applied for multiple comparisons when necessary. Categorical variables were analyzed using Pearson’s chi-square test or Fisher’s exact test, with continuity correction applied when appropriate. Logistic regression models were employed to assess associations between ultrasound characteristics and procedural outcomes. p values were calculated for all comparisons, with p values < 0.05 considered statistically significant.

## Results

A total of 468 patients with presumed RPOC were referred to OH.

### Preoperative findings

According to the pregnancy termination type, out of 199 patients, there were 160 (80.4%) cases after a medical TOP, 20 (10.1%) cases after a surgical TOP, 13 (6.5%) cases after a medical and surgical TOP, and 6 (3%) cases after a spontaneous miscarriage without any previous intervention. According to the type of delivery, there were 220 (81.8%) patients out of 269 cases after vaginal delivery and 49 (18.2%) patients after cesarean section.

Demographic data and preoperative findings are shown in Table 1.

**Table 1.** Demographic data and preoperative findings

	Minimum	Maximum	Mean	SD	n
Age (years)	19.0	49.7	32.9	5.2	468
TOP gestational age (weeks)	5.4	19.0	9.5	4.19	136
Delivery gestational age (weeks)	22.6	41.3	39.7	3.5	246
Time from TOP to procedure (weeks)	5.0	27.9	9.7	4.1	173
Time from delivery to procedure (weeks)	5.0	50.4	9.4	10.5	243
Parity (n)	0	5	1.3	0.9	467
RPOC thickness on US (mm)	3	30	10.8	5.0	267
RPOC length on US (mm)	5	95	18.4	10.1	166

*SD* standard deviation, *TOP* termination of pregnancy, *n* number, *RPOC* retained product of conception, *US* ultrasound

In 21 (4.5%) cases out of all 468 patients, removal of RPOC with a previous D&C was attempted but resulted in incomplete removal of RPOC.

Out of the 124 cases with available data, Doppler US showed no tissue vascularization in 104 (83.9%) patients, while predominately mild vascularization was observed in 20 (16.1%) patients.

Out of the 65 cases with available data, the shape of the borders between RPOC and the myometrium on US was described as smooth in 46 (70.8%) patients, unclear in 10 (15.4%) patients, and creased in 9 (13.8%) patients.

### Procedure outcomes

#### OH visual size estimation

Out of all 468 cases, 333 (71.2%) patients underwent OH removal, while the remaining 135 (28.8%) patients underwent diagnostic OH as no relevant RPOC was identified. In 304 (65.0%) out of 468 cases, only a small amount of RPOC based on OH estimation was visualized, while in 75 (16.0%) cases, a large amount of RPOC was seen. In the TOP group, consisting of 199 patients in 58 (29.2%) cases, OH revealed no relevant RPOC, 123 (61.8%) cases had a small amount of RPOC, while 18 (9.0%) cases had a large amount of RPOC. In the delivery group, consisting of 269 patients, in 31 (11.5%) cases, OH revealed no relevant RPOC, in 181 (67.3%) cases, a small amount of RPOC, while 57 (21.2%) cases had a large amount of RPOC (Table 2).

**Table 2.** Retained product of conception size estimation during office hysteroscopy based on pregnancy outcome

	TOP group	Delivery group	Total
Patients	199	269	468
No RPOC (*n*, %)	58 (29.2%)	31 (11.5%)	89 (19.0%)
Small RPOC (*n*, %)	123 (61.8%)	181 (67.3%)	304 (65.0%)
Large RPOC (*n*, %)	18 (9.0%)	57 (21.2%)	75 (16.0%)

*RPOC* retained product of conception, *TOP* termination of pregnancy

#### Procedure duration and pain estimation

The average duration of the procedure was 12.3 (5–70) min, 11.2 min for the TOP group and 13.4 min for the delivery group. The average pain score according to VAS was 2.1 ± 1.2. In the TOP group, the mean pain score was 2.5 ± 1.2, and in the delivery group, it was 1.8 ± 1.1.

#### Complications

Complications during the procedure were described in 103 (22.0%) cases. “Poor visibility” due to mild to moderate bleeding (up to 50–200 ml of estimated blood loss) or edema was present in 71 (15.2%) cases, of whom 7 (1.5%) patients received uterotonics. In 23 (4.9%) cases, “strong adherence of tissue” was reported, and in 9 (1.9%) cases, vasovagal reaction followed the procedure. No fluid overload was noted in any of the procedures. No uterine perforations occurred. All complications were managed conservatively and were classified as <grade II according to the Clavien–Dindo classification of surgical complications.

#### Completion of procedures and further management

Out of the 333 patients who underwent OH removal, the procedure was successfully completed in 225 (67.6%) cases as all the tissue was removed. Among these, 86 (38.2%) underwent TOP and 139 (61.8%) had deliveries; 15 (6.7%) had large RPOC, while 210 (93.3%) had small RPOC. Of the 108 incomplete OH removals, 35 (32.4%) underwent TOP and 73 (67.6%) had deliveries; 39 (36.1%) had large RPOC, while 69 (63.9%) had small RPOC.

Out of 108 patients in whom RPOC removal was assessed as incomplete, 36 (7.7%) were advised to undergo follow-up US imaging and conservative management, while 72 (15.4%) were further referred to operative hysteroscopy (50 cases, 10.7%) or D&C (22 cases, 4.7%).

### Analysis of possible limiting factors

#### Successfully complete OH removal vs incomplete OH removal

The pregnancy outcomes (TOP vs delivery) did not show a statistically significant correlation with the success of the OH removal (*p* = 0.302). Similarly, gestational age was not a significant factor in either the TOP group (*p* = 0.362) or the delivery group (*p* = 0.553).

**Fig. 1:**
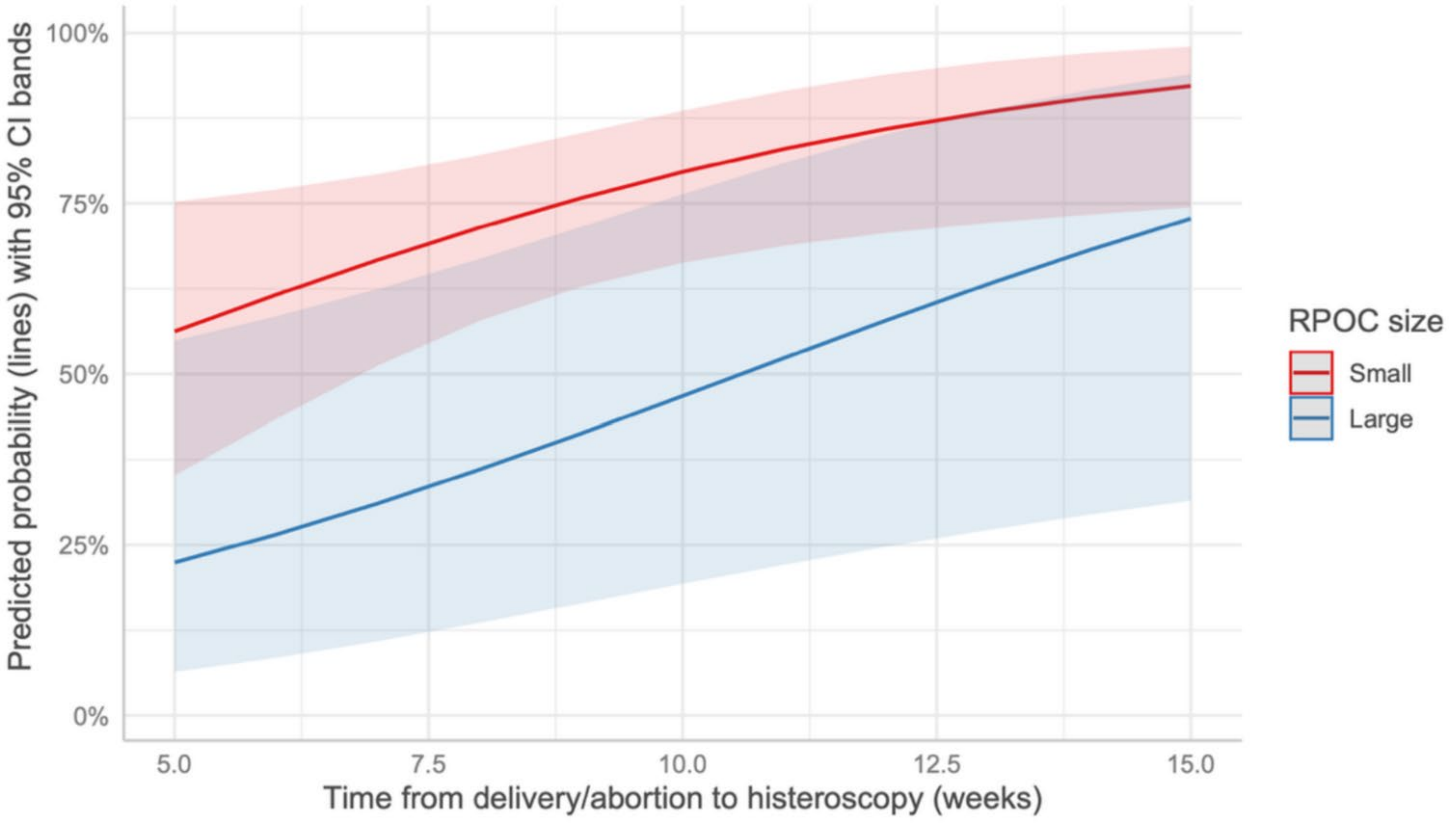
Predicted probability of complete removal in the TOP group based on the time from the end of pregnancy to OH

**Fig. 2:**
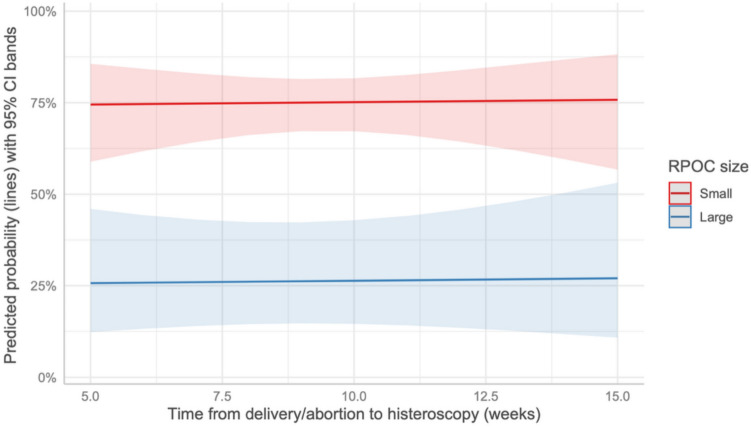
Predicted probability of complete removal in the delivery group based on the time from the end of pregnancy to OH

Conversely, prolonged time from the end of pregnancy to OH was a statistically significant factor for the complete removal of RPOC for patients in the TOP group (*p* = 0.025) (Fig. 1). In contrast, this factor was not significant for patients in the delivery group (*p* = 0.801) (Fig. 2).

Higher parity was statistically significant only in the delivery group (*p* < 0.007), including when only large RPOC procedures were analyzed (*p* = 0.034), but it was not significant in the TOP group (*p* = 0.218).

The success rate of the procedure was significantly higher in cases with small RPOC in both the TOP (*p* = 0.040) and delivery (*p* < 0.001) groups.

The duration of the procedure was significantly longer in cases of failure (*p* < 0.001).

The US characteristics, i.e., thickness, length, presence of vascularization, and type of RPOC–myometrial border, did not show a significant association with the completion of the procedure (*p* = 0.397, *p* = 0.874, *p* = 0.370, and *p* = 0.538, respectively).

#### Presence vs absence of RPOC on OH

**Fig. 3:**
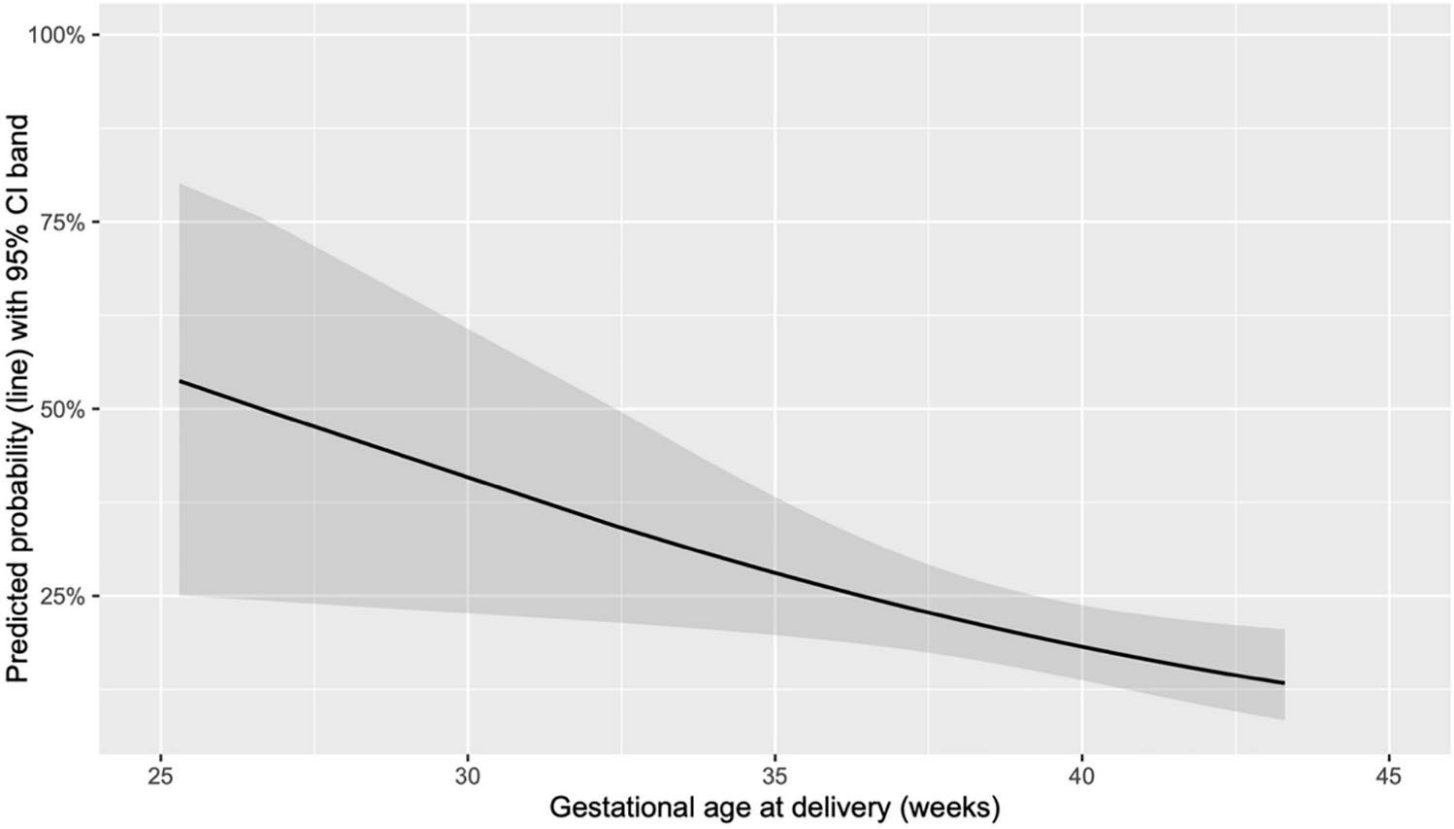
Predicted probability of absence of RPOC on OH based on the gestational age at delivery

**Fig. 4:**
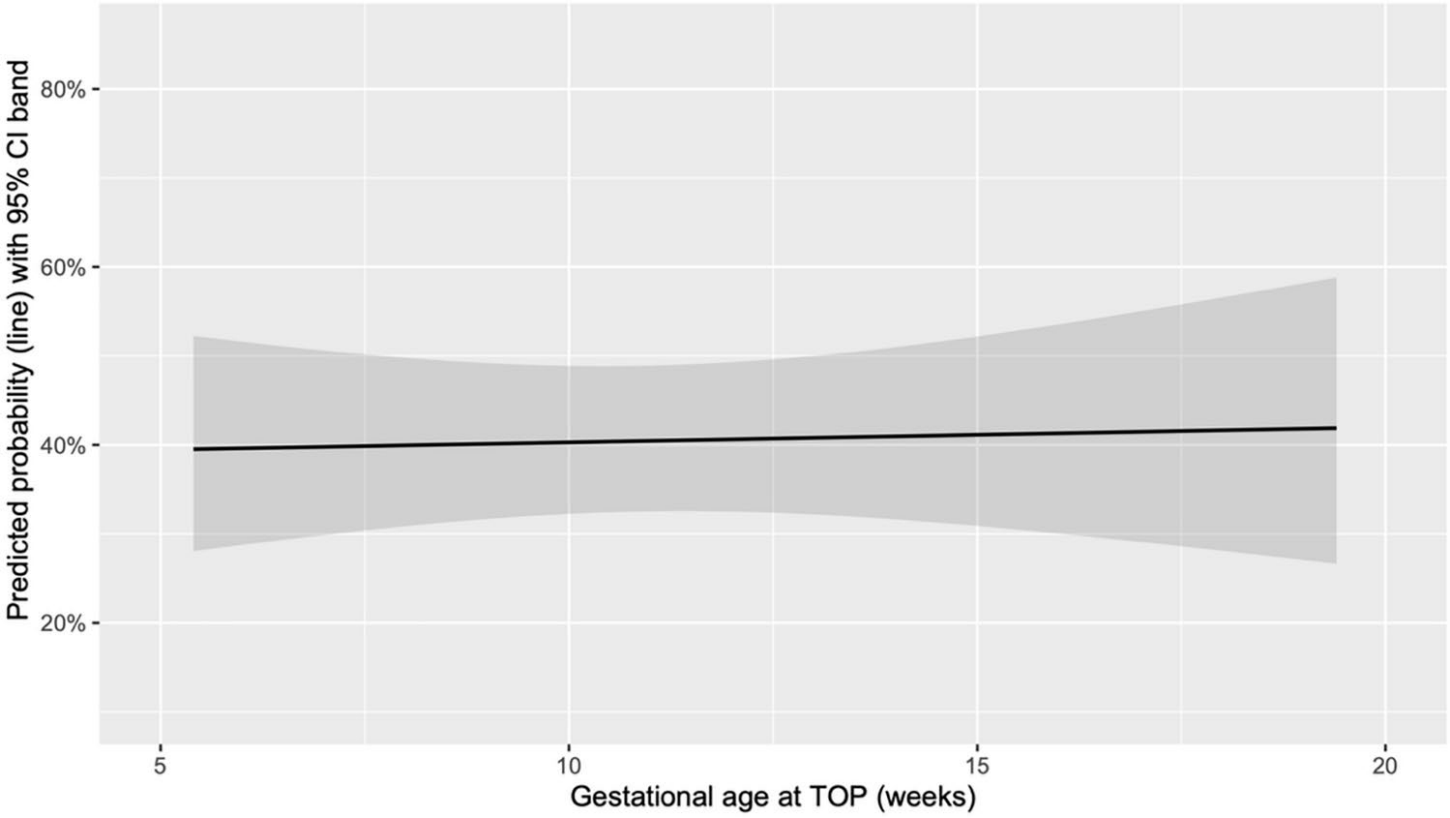
Predicted probability of absence of RPOC on OH based on the gestational age at TOP

The absence of RPOC during OH was significantly associated with higher gestational age only in deliveries (*p* = 0.003) (Fig. 3), while no significant difference was observed in cases of TOP (*p* = 0.846) (Fig. 4).

**Fig 5:**
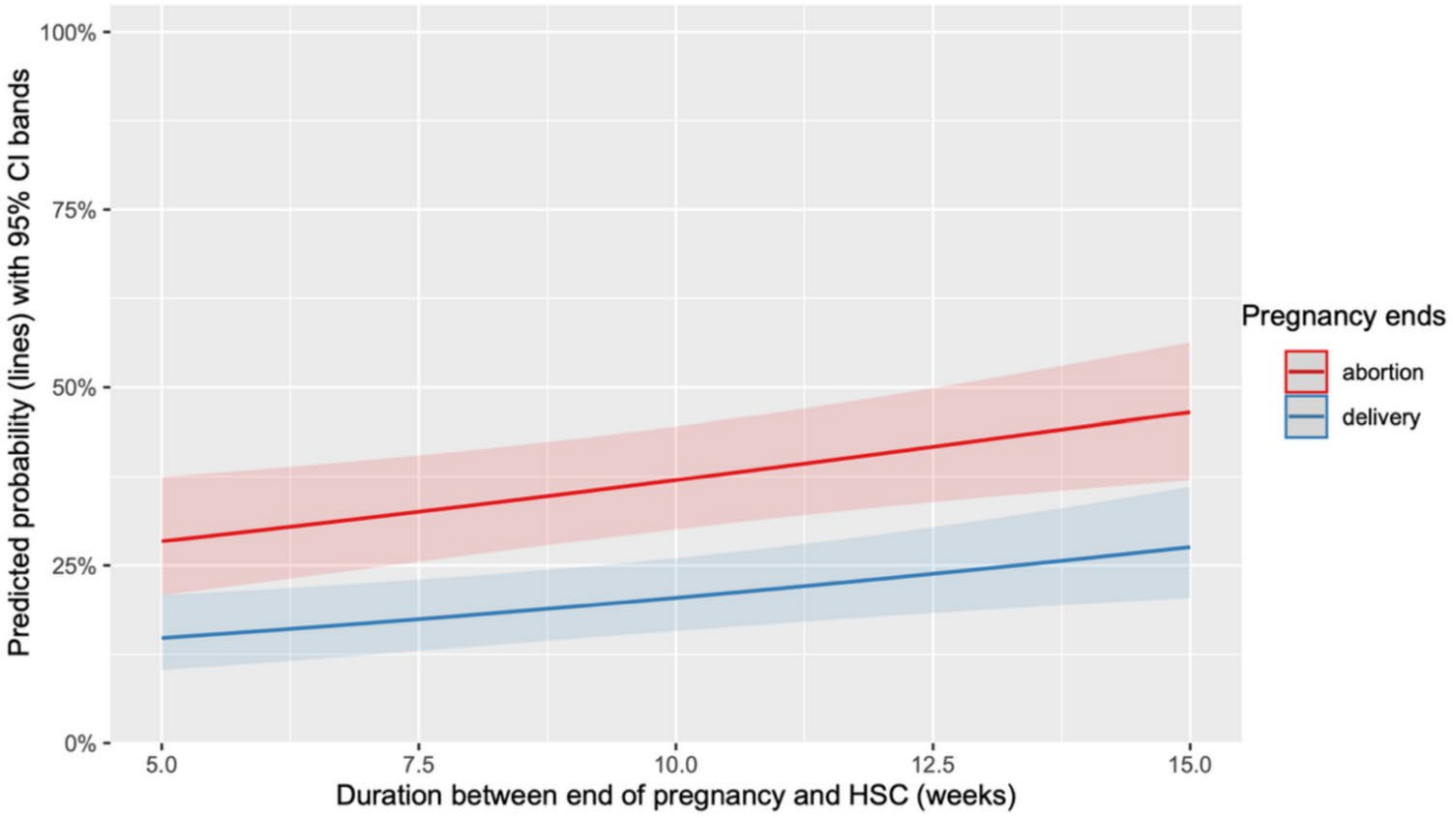
Predicted probability of absence of RPOC on OH based on the time from the end of pregnancy to the procedure

The time from the end of pregnancy to OH was significantly longer in cases of non-visualized RPOC for both TOP (*p* = 0.013) and deliveries (*p* = 0.003) (Fig. 5)

This outcome was more likely to occur when RPOC followed TOP (*p* < 0.001).

Parity did not show a significant correlation (*p* = 0.117).

US thickness and length were found to be lower in cases of OH in which no RPOC were visualized (*p* = 0.007 and *p* = 0.011, respectively), while the presence of vascularization and the type of RPOC–myometrial border on US did not show an association (*p* = 0.243 and *p* = 0.227, respectively).

## Discussion

To the best of our knowledge, this is the first study to analyze the factors associated with incomplete removal and the absence of intrauterine remnants during OH performed in patients with suspected RPOC.

### Procedure outcomes

#### Procedure duration and pain estimation

The average duration of the procedure was relatively short (12.3 minutes), and notably lower than the time reported by Raz et al., which was 19.1 minutes. Moreover, these authors demonstrated that procedure times were comparable between the see-and-treat approach and operative hysteroscopy under general anesthesia [21].

It is important to acknowledge that OH may not be feasible for all patients as pain (primarily caused by the introduction of the hysteroscope through a non-dilated cervical canal) can be a substantial limitation for some women. Various pain management strategies have been proposed for office-based operative hysteroscopies, including preoperative oral analgesia, inhaled analgesia (e.g., nitrous oxide), local intracervical or intramyometrial anesthesia, paracervical blocks, and intravenous sedation [22]. Each method has distinct advantages and disadvantages, and no single approach has been universally recognized as superior or optimal [23]. Nevertheless, the findings of this study indicate that the removal of small RPOC can be effectively performed during OH with minimal pain or discomfort for patients; average VAS was 2.1, even in the absence of anesthesia or analgesia. This is particularly significant for specific patient populations, such as breastfeeding mothers, for whom a quick discharge and avoidance of anesthesia are critical.

#### Complications

The safety of OH has been well-established as complications during the procedure were rare and all were managed conservatively. Bleeding was the most common complication; however, it was predominantly self-limiting, as uterotonic agents were required only in 1.5% of all patients who had OH. It is important to note that patients with strong tissue vascularization identified in the US were excluded from this study. This is consistent with findings by Alonso Pacheco et al. [24], who demonstrated that many cases with strong tissue vascularization required the use of monopolar energy during the procedure to mitigate the risk of uncontrollable massive bleeding and were deemed more appropriate for operative room settings. Also, Sonier et al. [25] reported a higher rate of bleeding during OH, with 4% of patients with RPOC requiring embolization. This may be attributed to the lack of preoperative vascularization assessment via US in their study. Conversely, Raz et al. [21] reported a lower bleeding rate than the present study. A plausible explanation for this difference is that Raz et al. used a smaller cut-off size of 2 cm for RPOC, whereas this study included cases with RPOC up to 3 cm in size, which may inherently carry a higher risk of bleeding. Furthermore, they reported only episodes of excessive bleeding, whereas in this study, any bleeding compromising hysteroscopic visibility was recorded, leading to a broader definition of bleeding-related events. No cases of fluid overload syndrome were reported in this study, highlighting the advantage of using a physiologic saline solution as a distension medium over non-physiologic, electrolyte-free solutions commonly employed in monopolar resectoscopy. Some authors [26] have suggested that the see-and-treat approach in OH is inherently safer than operative hysteroscopy under general anesthesia. This reduced risk of complications may be attributed to the procedure being performed on awake patients, who can provide immediate feedback in case of discomfort or pain. This awareness can help prevent serious complications, such as uterine perforation or excessive bleeding, by allowing the operator to adjust their technique promptly [17].

#### Completion of procedures and further management

The see-and-treat approach used in this study allowed treatment of RPOC in a single session, with only a minority of cases deemed incomplete requiring a follow-up procedure, especially when small RPOC were involved. This result aligns with the findings of Perez-Medina et al. [27], who reported a 91.8% success rate for RPOC removal using OH. This study also confirmed that more than one-quarter of OH did not reveal any RPOC, highlighting a non-negligible rate of cases without intrauterine pathology. This is partly due to the minimally invasive nature of the procedure, which does not require general anesthesia and can be performed in an outpatient setting with minimal discomfort for the patient. Therefore, in cases where US findings were inconclusive, clinicians preferred to proceed with diagnostic OH that provides a definitive diagnosis in a single procedure, avoiding the prolonged uncertainty, treatment delays, and psychological burden associated with serial US assessments. However, to optimize patient management and reduce both the number of procedures without RPOC and repetitive US evaluations, there is a clear need for more precise and standardized US diagnostic criteria that can reliably define true RPOC.

### Analysis of possible limiting factors

#### Successfully complete OH removal vs incomplete OH removal

The time from the end of pregnancy to the OH procedure was a significant factor in determining successful removal in the TOP group. Interestingly, this factor also proved significant in the delivery group, but only when analyzing large RPOC cases. This finding supports the hypothesis that a longer interval after pregnancy might facilitate tissue organization and reduce tissue vascularization, making the procedure more likely to succeed.

Notably, this observation differs from the findings of Alonso Pacheco et al. [24], who reported no significant differences in procedural outcomes related to the time elapsed since the end of pregnancy.

RPOC size and procedure duration were also significant predictors of incomplete removal. Larger RPOC and more prolonged procedures were more frequently associated with failure, consistent with prior studies by Cohen et al. [28] and Mohr-Sasson et al. [29], who identified RPOC size as a major contributor to procedure failure during OH. Larger masses are more likely to present technical challenges due to their dimensions or stronger myometrial adhesion, increasing the risk of incomplete removal.

Parity was a significant factor in the delivery group, even when only cases involving large RPOC were considered, but it was not statistically significant in the TOP group. This difference may be explained by the physiological changes associated with parity and delivery. In multiparous women, the cervix is often more dilated, and the uterine cavity larger, potentially facilitating the removal of larger masses. Conversely, in the TOP group, where parity is often lower, these anatomical adaptations may be less pronounced, diminishing the influence of parity on the procedural outcome.

Despite these findings, other factors did not show statistically significant associations with successful OH removal. A distinction might reasonably be expected between RPOC following TOP and those occurring postpartum, particularly after vaginal delivery. In the latter case, a dilated cervix and a larger uterine cavity could facilitate the removal of larger masses. Similarly, one might anticipate a higher likelihood of procedural failure in connection with gestational age. In the TOP group, more advanced gestations are associated with deeper placental implantation and larger RPOC, and vice versa. In the delivery group, lower gestations are associated with deeper placental implantation and stronger adhesions to the myometrium. Surprisingly, however, statistical analysis did not confirm these assumptions. This unexpected result suggests that other factors may play a significant role in determining procedural success.

Lastly, US criteria alone did not appear to be reliable predictors of procedural outcome. While the US remains an essential diagnostic tool for identifying RPOC, its reliability varies significantly across studies [8, 9]. Additionally, endometrial thickness cut-off values for diagnosing RPOC remain poorly defined. Some authors have proposed a threshold of 15 mm [30]. Conversely, Sawyer et al. [31] evaluated the predictive value of endometrial thickness for RPOC and found no specific cut-off that could reliably confirm its presence. This conclusion is further supported by Levin et al. [32], who demonstrated that physician interpretation of RPOC characteristics based on direct hysteroscopic visualization is a stronger predictor for RPOC removal than clinical parameters or US findings alone. These findings also support the present study. Nonetheless, accurate US evaluation remains a cornerstone of the diagnostic process [33]. Assessing mass vascularization with Doppler is particularly important as it helps physicians perform a precise preoperative evaluation of bleeding risk. Moreover, differentiating between highly vascular RPOC and acquired uterine arteriovenous malformation (AVM) is essential, given the distinct treatment approaches required. Unlike AVM, where vascularization typically extends into the myometrial layer, RPOC vascularization is confined to the endometrial layer [34]. When AVM is suspected, further imaging, such as computed tomography angiography followed by arteriography, is necessary to confirm the diagnosis and guide potential embolization treatment [35]. Given that there were no major bleedings requiring interventions other than the occasional application of uterotonics in this study, the US evaluation of RPCO vascularization with Doppler was correct. However, the limited predictive value of US in confirming the presence of RPOC or anticipating the completeness of their removal has practical implications for clinical management. In particular, it suggests that US findings alone may not be sufficient for optimal triage of patients to OH. The proportion of cases in which no RPOC were ultimately visualized reflects the diagnostic challenge of interpreting nonspecific or borderline US features. To enhance clinical decision-making, a more comprehensive approach may be considered, integrating US results with clinical variables, such as time elapsed since pregnancy, parity, and gestational age. In some cases, repeated imaging after the first menstrual cycle may help differentiate between evolving physiological changes and persistent RPOC. Such strategies could support a more refined patient selection process and contribute to improved procedural planning.

#### Presence vs absence of RPOC on OH

The absence of RPOC during OH was significantly associated with gestational age in the delivery group but not in the TOP group.

This difference may be attributed to physiological and anatomical changes postpartum. After delivery, particularly vaginal delivery, the uterine cavity is larger, and the cervix remains dilated for some time, potentially allowing easier natural expulsion of RPOC. In contrast, in TOP cases, these changes are less pronounced, and the uterine environment may not as readily facilitate spontaneous expulsion, leading to a less apparent relationship with gestational age.

The time elapsed from the end of pregnancy to OH was significantly longer in cases of absence of RPOC during OH for both TOP and deliveries. This finding underscores the importance of allowing sufficient time for potential spontaneous expulsion of RPOC, especially in case of unclear US findings, as RPOC may resolve without intervention. National protocols recommend performing OH eight weeks after delivery and after the second menstrual period following TOP. In the TOP group, suspected RPOCs are initially evaluated by the US after the first menstruation, while any eventual OH removal is scheduled after the second menstruation. This protocol may have favored the spontaneous resolution of smaller RPOC in some patients, supporting a conservative approach in select cases. Accordingly, a watchful waiting strategy is advisable before OH evaluation, particularly without symptoms or significant clinical findings.

In the present study, the absence of RPOC more likely occurred following TOP. This could be explained by the more frequent use of the US for follow-up in these cases and a lower threshold for intervention based on imaging findings, even when asymptomatic. In contrast, postpartum management may rely more on clinical symptoms, leading to fewer unwarranted procedures.

As expected, US findings, such as lower RPOC thickness and length, were significantly associated with cases in which no RPOC were visualized during OH. This highlights the need for improved diagnostic criteria to set the cut point for cases requiring intervention and those likely to resolve spontaneously.

This study demonstrates several strengths. It was conducted at a single center with an experienced team. The inclusion of a large patient cohort and the application of standardized protocols enhance the study’s validity. Despite these strengths, the study is not without limitations. The retrospective design inherently introduces potential selection and information biases. The absence of randomization further limits the ability to control confounding variables, which may have influenced the results. Additionally, this study excluded patients with strongly vascularized tissue or endometrial thickness greater than 30 mm, restricting the findings to a specific subset of cases and limiting their generalizability to higher-risk scenarios. Moreover, some US variables were missing in a substantial portion of cases, which may have limited the statistical power of related analyses and introduced potential information bias. Further research, particularly prospective and randomized controlled trials, is essential to validate these findings, expand the applicability of OH to more diverse clinical settings, and establish standardized guidelines for its optimal use.

## Conclusions

This study highlights key factors associated with incomplete RPOC removal and procedures performed for suspected RPOC in which no intrauterine remnants were ultimately confirmed during OH. Incomplete removal was associated with larger RPOC and consequently prolonged procedures, while the absence of RPOC was linked to insufficient waiting periods post-pregnancy and suboptimal diagnostic criteria. These findings emphasize the need for refined patient selection, standardized US criteria, and careful preoperative planning to optimize outcomes. While limitations exist, this study provides a foundation for future research to enhance the efficacy of OH in managing RPOC.

## Data Availability

No datasets were generated or analysed during the current study.

## References

[1] Westendorp IC, Ankum WM, Mol BW, Vonk J (1998) Prevalence of Asherman’s syndrome after secondary removal of placental remnants or a repeat curettage for incomplete abortion. Hum Reprod. 13:3347–50 9886512 10.1093/humrep/13.12.3347

[2] Smorgick N, Barel O, Fuchs N, Ben-Ami I, Pansky M, Vaknin Z (2014) Hysteroscopic management of retained products of conception: meta-analysis and literature review. Eur J Obstet Gynecol Reprod Biol. 173:19–22 24332096 10.1016/j.ejogrb.2013.11.020

[3] Hamerlynck TW, Blikkendaal MD, Schoot BC et al (2013) An alternative approach for removal of placental remnants: hysteroscopic morcellation. J Minim Invasive Gynecol. 20(6):796–802 24183271 10.1016/j.jmig.2013.04.024

[4] Achiron R, Goldenberg M, Lipitz S, Mashiach S (1993) Transvaginal duplex Doppler ultrasonography in bleeding patients suspected of having residual trophoblastic tissue. Obstet Gynecol. 81(4):507–11 8459957

[5] Alcázar JL (1998) Transvaginal ultrasonography combined with color velocity imaging and pulsed Doppler to detect residual trophoblastic tissue. Ultrasound Obstet Gynecol. 11(1):54–8 9511197 10.1046/j.1469-0705.1998.11010054.x

[6] Van den Bosch T, Van Schoubroeck D, Timmerman D (2015) Maximum peak systolic velocity and management of highly vascularized retained products of conception. J Ultrasound Med 34:1577–1582 26254150 10.7863/ultra.15.14.10050

[7] Budorick NE, Figueroa R, Vizcarra M, Shin J (2016) Another look at ultrasound and magnetic resonance imaging for diagnosis of placenta accreta. J Maternal-Fetal Neonatal Med. 24:1–6 10.1080/14767058.2016.1252744 27806657

[8] Ben-Ami I, Schneider D, Maymon R, Vaknin Z, Herman A, Halperin R (2005) Sonographic versus clinical evaluation as predictors of residual trophoblastic tissue. Hum Reprod. 20:1107 15650045 10.1093/humrep/deh689

[9] Zalel Y, Cohen SB, Oren M, Seidman DS, Zolti M, Achiron R, Goldenberg M (2001) Sonohysterography for the diagnosis of residual trophoblastic tissue. J Ultrasound Med. 20:877 11503924 10.7863/jum.2001.20.8.877

[10] Ben-Ami I, Melcer Y, Smorgick N, Schneider D, Pansky M, Halperin R (2014) A comparison of reproductive outcomes following hysteroscopic management versus dilatation and curettage of retained products of conception. Int J Gynecol Obstet 127(1):86–9 10.1016/j.ijgo.2014.05.003 24997472

[11] Cohen SB, Kalter-Ferber A, Weisz BS, Zalel Y, Seidman DS, Mashiach S et al (2001) Hysteroscopy may be the method of choice for management of residual trophoblastic tissue. J Am Assoc Gynecol Laparosc 8(2):199–202 11342724 10.1016/s1074-3804(05)60577-4

[12] Gulisano M, Gulino FA, Incognito GG, Cimino M, Dilisi V, Di Stefano A, D’Urso V, Cannone F, Martire FG, Palumbo M (2023) Role of Hysteroscopy on Infertility: the eternal dilemma. Clin Exp Obstet Gynecol 50(5):99

[13] Centini G, Troia L, Lazzeri L, Petraglia F, Luisi S (2016) Modern operative hysteroscopy. Minerva Ginecol. 68(2):126–32 26930389

[14] Di Guardo F, Incognito GG, Lello C, D’Urso G, Genovese F, Palumbo M (2022) Efficacy of sonohysterography and hysteroscopy for evaluation of endometrial lesions in tamoxifen treated patients: a systematic review. Eur J Gynaecol Oncol 43(1):78–86

[15] ACOG Technology Assessment No (2018) 13: hysteroscopy. Obstet Gynecol. 131(5):e151–e6 10.1097/AOG.0000000000002634 29683912

[16] D’Urso V, Gulino FA, Incognito GG et al (2023) Hysteroscopic findings and operative treatment: all at once? J Clin Med. 12(13):4232 37445266 10.3390/jcm12134232 PMC10342816

[17] Bettocchi S, Ceci O, Nappi L, Di Venere R, Masciopinto V, Pansini V et al (2004) Operative office hysteroscopy without anesthesia: analysis of 4863 cases performed with mechanical instruments. J Am Assoc Gynecol Laparosc. 11:59 15104833 10.1016/s1074-3804(05)60012-6

[18] Gambadauro P, Martınez-Maestre MA, Torrejòn R (2014) When is see-andtreat hysteroscopic polypectomy successful? Eur J Obstet Gynecol Reprod Biol. 178:70–73 24792666 10.1016/j.ejogrb.2014.03.048

[19] Jakopič Maček K, Blaganje M, Kenda Šuster N, Drusany Starič K, Kobal B (2020) Office hysteroscopy in removing retained products of conception - a highly successful approach with minimal complications. J Obstet Gynaecol. 40(8):1122–1126 31793362 10.1080/01443615.2019.1679736

[20] Leone FPG, Timmerman D, Bourne T, Valentin L, Epstein E, Goldstein SR et al (2010) Terms, definitions, and measurements to describe the sonographic features of the endometrium and intrauterine lesions: a consensus opinion from the international endometrial tumor analysis (IETA) group. Ultrasound Obstet Gynecol 35:103–112 20014360 10.1002/uog.7487

[21] Raz N, Sigal E, Gonzalez Arjona F et al (2022) See-and-treat in-office hysteroscopy versus operative hysteroscopy for the treatment of retained products of conception: a retrospective study. J Obstet Gynaecol Res. 48(9):2459–2465 35698805 10.1111/jog.15327 PMC9541046

[22] Salazar CA, Isaacson KB (2018) Office operative hysteroscopy: an update. J Minim Invasive Gynecol. 25:199–208 28803811 10.1016/j.jmig.2017.08.009

[23] Vitale SG, Alonso Pacheco L, Haimovich S et al (2021) Pain management for in-office hysteroscopy a practical decalogue for the operator. J Gynecol Obstet Hum Reprod. 50:101976 33166706 10.1016/j.jogoh.2020.101976

[24] Alonso Pacheco L, Timmons D, Saad Naguib M, Carugno J (2019) Hysteroscopic management of retained products of conception: a single center observational study. Facts Views Vis Obgyn. 11:217–222 32082527 PMC7020944

[25] Sonnier L, Torre A, Broux P, Fauconnier A, Huchon C (2017) Evaluation of fertility after operative hysteroscopy to remove retained products of conception. Eur J Obstet Gynecol Reprod Biol 211:98–102 28214435 10.1016/j.ejogrb.2017.02.003

[26] Bennett A, Lepage C, Thavorn K, Fergusson D, Murnaghan O, Coyle D et al (2019) Effectiveness of outpatient versus operating room hysteroscopy for the diagnosis and treatment of uterine conditions: a systematic review and meta-analysis. J Obstet Gynaecol Can. 41:930–41 30528838 10.1016/j.jogc.2018.10.002

[27] Pérez-Medina T, Sancho-Saúco J, Ríos M et al (2014) Hysteroscopy in pregnancy-related conditions: descriptive analysis in 273 patients. J Minim Invasive Gynecol. 21(3):417–425 24280360 10.1016/j.jmig.2013.11.004

[28] Cohen A, Cohen Y, Sualhi S, Rayman S, Azem F, Rattan G (2017) Office hysteroscopy for removal of retained products of conception: can we predict treatment outcome? Clin Exp Obstet Gynecol. 44(5):683–685

[29] Mohr-Sasson A, Gur T, Meyer R, Mashiach R, Stockheim D (2022) Office operative hysteroscopy for the management of retained products of conception. Reprod Sci. 29(3):761–767 35020188 10.1007/s43032-022-00849-7

[30] Rein DT, Schmidt T, Hess AP et al (2011) Hysteroscopic management of residual trophoblastic tissue is superior to ultrasound-guided curettage. J Minim Invasive Gynecol. 18(6):774–778 22024264 10.1016/j.jmig.2011.08.003

[31] Sawyer E, Ofuasia E, Ofili-Yebovi D, Helmy S, Gonzalez J, Jurkovic D (2007) The value of measuring endometrial thickness and volume on transvaginal ultrasound scan for the diagnosis of incomplete miscarriage. Ultrasound Obstet Gynecol. 29:205 17201018 10.1002/uog.3914

[32] Levin I, Almog B, Ata B, Ratan G, Many A (2010) Clinical and s graphic findings in suspected retained trophoblast after pregnancy do not predict the disorder. J Minim Invasive Gynecol 17:66 20129335 10.1016/j.jmig.2009.11.002

[33] Scribner D, Fraser R (2016) Diagnosis of acquired uterine arteriovenous malformation by Doppler ultrasound. J Emerg Med 51:168–71 27260690 10.1016/j.jemermed.2016.04.028

[34] Wada Y, Takahashi H, Suzuki H et al (2021) Expectant management of retained products of conception following abortion: a retrospective cohort study. Eur J Obstet Gynecol Reprod Biol. 260:1–5 33689917 10.1016/j.ejogrb.2021.02.028

[35] De Winter J, De Raedemaecker H, Muys J, Jacquemyn Y (2017) The value of postpartum ultrasound for the diagnosis of retained products of conception: a systematic review. Facts Views Vis Obgyn 9:207–16 30250654 PMC6143083

